# Supplementary material for: Quality of Life in Women after Deep Endometriosis Surgery: Comparison with Spanish Standardized Values
Source: J Clin Med. 2022 Oct 20;11(20):6192. doi: 10.3390/jcm11206192 (PMC9605478; doi:10.3390/jcm11206192)
Supplement: Supplementary file 1 [file jcm-11-06192-s001.zip › jcm-1941558-supplementary.pdf]

Journal of Clinical Medicine

Table S1. Correlations between the dimensions of SF-12 in the cohort.

	1	2	3	4	5	6	7	8	9
**1. Physical function**	1.00								
**2. Physical role**	-0.71*	1.00							
**3. Body pain**	-0.76*	0.80*	1.00						
**4. Social function**	0.69*	-0.75*	-0.74*	1.00					
**5. Emotional role**	-0.57*	0.70*	0.66*	-0.57*	1.00				
**6. Mental health**	-0.64*	0.63*	0.63*	-0.55*	0.61*	1.00			
**7. Vitality**	-0.63*	0.58*	0.53*	-0.59*	0.50*	0.57*	1.00		
**8. General health**	-0.70*	0.67*	0.72*	-0.64*	0.55*	0.60*	0.62*	1.00	
**9. Physical component**	-0.37*	0.73*	0.71*	-0.56*	0.27	0.25	0.37*	0.67*	1.00
**10. Mental component**	-0.55*	0.47*	0.45*	-0.31*	0.82*	0.78*	0.60*	0.45*	-0.06

Data show the Spearman´s correlation (r). The * shows *p*-Value <0.05.

Table S2. Dimensions and components of SF-12 among women with or without bowel nodule resection.

	No (n=32)	Discoid (n=12)	Segmental (n=40)	Shaving (n=28)	*p*-Value
Physical function	20.3 (30.7)	14.6 (22.5)	18.1 (28.3)	25.9 (31.5)	0.640
Physical role	51.6 (44.9)	50.0 (42.6)	62.5 (44.9)	48.2 (48.1)	0.570
Body pain	62.5 (43.1)	77.1 (34.5)	69.4 (39.8)	52.7 (42.7)	0.258
Social function	27.3 (37.2)	20.8 (38.2)	28.1 (38.5)	38.4 (40.0)	0.524
Emotional role	67.2 (39.4)	70.8 (45.0)	62.5 (40.4)	53.6 (47.0)	0.554
Mental health	55.9 (25.2)	62.0 (22.5)	61.1 (27.4)	59.9 (27.3)	0.833
Vitality	60.0 (32.5)	75.0 (25.8)	59.5 (30.9)	51.4 (31.0)	0.184
General health	48.1 (37.4)	53.8 (37.7)	55.2 (31.5)	46.1 (32.9)	0.685
Physical component	35.2 (20.4)	36.8 (19.5)	42.4 (16.4)	34.7 (22.0)	0.308
Mental component	60.4 (24.5)	66.6 (31.1)	58.1 (24.6)	57.0 (30.5)	0.748

Data show mean and standard deviation (SD). The *p*-Value was extracted using an independent one-way ANOVA test.

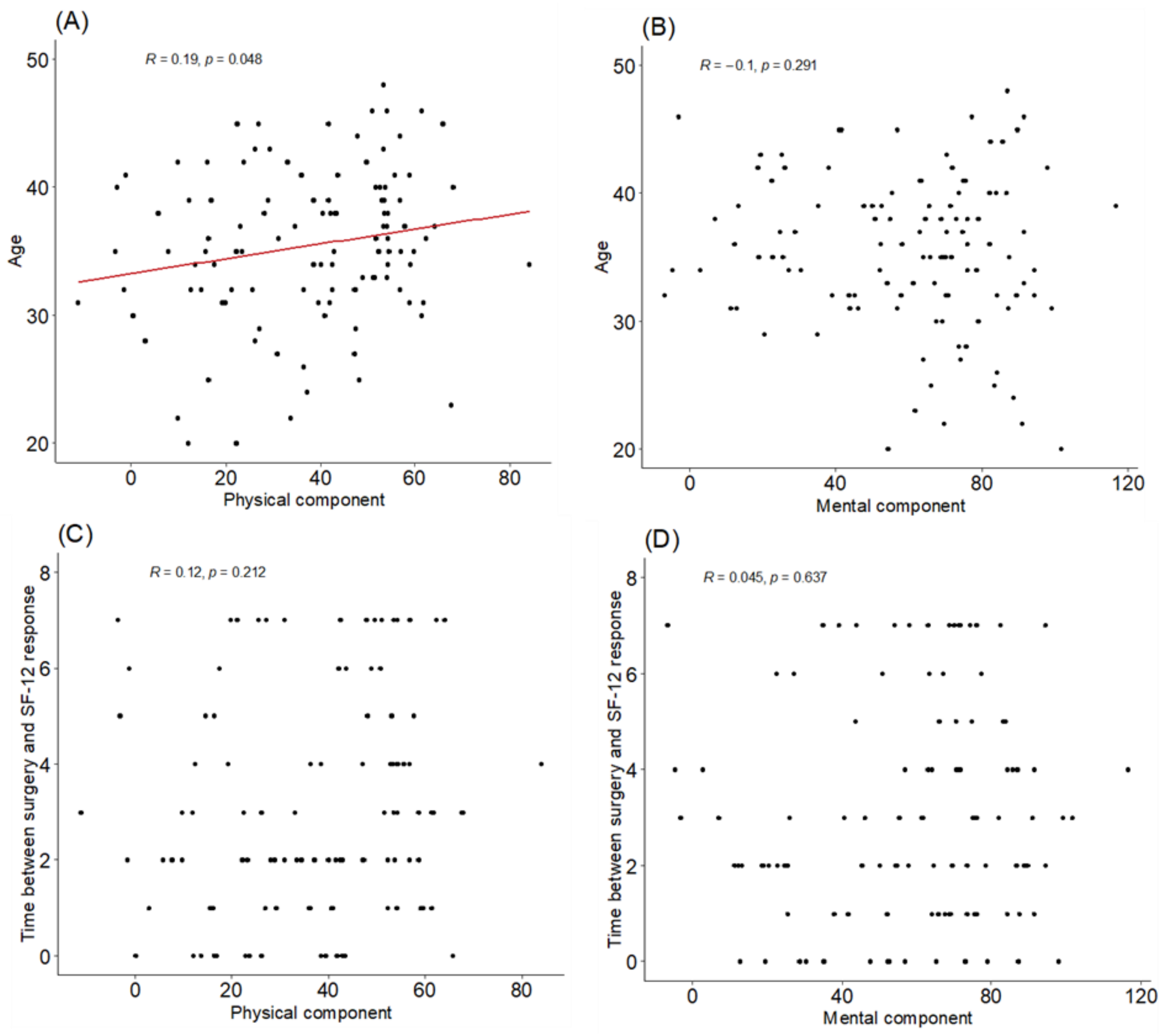

Figure S1. Scatter plots regarding women's age and physical component (**A**), mental component (**B**) of SF-12, time between surgery and SF-12 response and the scores of the physical component (**C**), and the scores of the mental component (**D**). Pearson´s coefficient (R) and associated *p*-value (*p*) are reported. In the significant correlation (p <0.05), the linear regression trend was reported in red.
